# Supplementary material for: Genome Characterization and Probiotic Potential of Corynebacterium amycolatum Human Vaginal Isolates
Source: Microorganisms. 2022 Jan 23;10(2):249. doi: 10.3390/microorganisms10020249 (PMC8878833; doi:10.3390/microorganisms10020249)
Supplement: Supplementary file 1 [file microorganisms-10-00249-s001.zip › Supplementary materials.pdf]

**Supplementary materials for "Genome characterization and probiotic potential of *Corynebacterium amycolatum* human vaginal isolates"**

Supplementary table S1. Comparison of genome characteristics of ICIS5, ICIS 9 and ICIS 53 with other *Corynebacterium amycolatum* strains

Supplementary table S2. eggNOG categories of coding proteins in *C. amycolatum* ICIS 5, ICIS 9 and ICIS 53 genomes

Supplementary table S3. Condensed Gene of ICIS 9

Supplementary table S4. Antibiotic resistant genes detected in *C. amycolatum* ICIS 5 and ICIS 9

Supplementary table S5. Virulence related genes detected in *C. amycolatum* ICIS 5, ICIS 9 and ICIS 53

Supplementary fig. S1. Proposed biosynthetic gene cluster of merochlorins A–D–like compound in the *C. amycolatum* ICIS 5. The most similar gene cluster from *Streptomyces sp.* CNH189 is shown, with related genes drawn in the same color to highlight inter-cluster rearrangements.

Supplementary fig. S2. Proposed biosynthetic gene cluster of merochlorins A–D–like compound in the *C. amycolatum* ICIS 9. The most similar gene cluster from *Streptomyces sp.* CNH189 is shown, with related genes drawn in the same color to highlight inter-cluster rearrangements.

Supplementary fig. S3. Proposed biosynthetic gene cluster of merochlorins A–D–like compound in the *C. amycolatum* ICIS 53. The most similar gene cluster from *Streptomyces sp.* CNH189 is shown, with related genes drawn in the same color to highlight inter-cluster rearrangements.

Supplementary fig. S4. Proposed biosynthetic gene cluster of phthoxazolin–like compound in the *C. amycolatum* ICIS 5. The most similar gene cluster from *Streptomyces avermitilis* is shown, with related genes drawn in the same color to highlight inter-cluster rearrangements.

Supplementary fig. S5. Proposed biosynthetic gene cluster of phthoxazolin–like compound in the *C. amycolatum* ICIS 53. The most similar gene cluster from *Streptomyces avermitilis* is shown, with related genes drawn in the same color to highlight inter-cluster rearrangements.
