# Supplementary material for: Genome Characterization and Probiotic Potential of Corynebacterium amycolatum Human Vaginal Isolates
Source: Microorganisms. 2022 Jan 23;10(2):249. doi: 10.3390/microorganisms10020249 (PMC8878833; doi:10.3390/microorganisms10020249)
Supplement: Supplementary file 1 [file microorganisms-10-00249-s001.zip › Table S1.pdf]

Supplementary Table S1. Comparison of genome characteristics of ICIS5, ICIS 9 and ICIS 53 with that of other *Corynebacterium amycolatum* strains

Strain	Isolation site	WGS Project	Sequence	Genome size (bp)	GC(%)	Assembly level	Genes (total)	Genes (coding)	CDSs (total)	Pseudo Genes (total)	rRNA	tRNA	CRISPRs
ICIS 5	vaginal swabs	SSOR01	115	2,474,151	58,80	Contig	2195	2062	2109	47	30	53	1
ICIS 9	vaginal swabs	MTPT01	181	2,587,830	58,60	Contig	2392	2277	2330	53	6	53	-
ICIS 53	vaginal swabs	MIFV01	41	2,460,257	59,00	Contig	2173	2076	2110	34	7	53	-
SK46*	human skin	ABZU01	48	2,513,912	58,60	Contig	2198	2098	2135	37	2	49	1
NCTC7243	human nose	UFXE01	6	2,638,280	58,80	Contig	2302	2166	2268	52	28	53	2
UMB0042	female urine	PKHS01	16	2,479,600	58,9	Scaffold	2184	2093	2125	32	4	52	-
UMB0338	female urine	PKHT01	39	2,465,259	58,9	Scaffold	2183	2068	2144	46	13	53	-
UMB7760	female urine	VYVQ01	65	2,422,276	58,9	Contig	2141	2045	2078	33	6	54	1
UMB9184	female urine	VYVF01	61	2,494,409	58,9	Contig	2206	2111	2142	31	7	54	1
UMB1182	female urine	VYWH01	41	2,425,544	58,7	Contig	2149	2040	2086	46	6	54	-
UMB1310	female urine	VYWB01	54	2,492,575	58,8	Contig	2215	2096	2153	57	5	54	-
UMB9256	female urine	VYVD01	65	2,565,104	58,8	Contig	2276	2166	2208	42	12	53	1

**C. amycolatum*, strain SK46 (HMP ID 0282) is a reference genome for The Human Microbiome Project (HMP).
