# Supplementary material for: Genome Characterization and Probiotic Potential of Corynebacterium amycolatum Human Vaginal Isolates
Source: Microorganisms. 2022 Jan 23;10(2):249. doi: 10.3390/microorganisms10020249 (PMC8878833; doi:10.3390/microorganisms10020249)
Supplement: Supplementary file 1 [file microorganisms-10-00249-s001.zip › Table S2.pdf]

Supplementary Table S2. eggNOG categories of coding proteins *in Corynebacterium amycolatum* ICIS 5, ICIS 9 and ICIS 53 genomes.

Class	Name	ICIS 5		ICIS 9		ICIS 53	
J	Translation, ribosomal structure and biogenesis	141	6,84 (%)	141	6,19 (%)	136	6,55 (%)
A	RNA processing and modification	1	0,05 (%)	1	0,04 (%)	1	0,05 (%)
K	Transcription	99	4,80 (%)	101	4,44 (%)	97	4,67 (%)
L	Replication, recombination and repair	130	6,30 (%)	228	10,01 (%)	100	4,82 (%)
B	Chromatin structure and dynamics	0	0,00 (%)	0	0,00 (%)	0	0,00 (%)
D	Cell cycle control, cell division, chromosome partitioning	19	0,92 (%)	19	0,83 (%)	21	1,01 (%)
Y	Nuclear structure	0	0,00 (%)	0	0,00 (%)	0	0,00 (%)
V	Defense mechanisms	48	2,33 (%)	50	2,20 (%)	44	2,12 (%)
T	Signal transduction mechanisms	48	2,33 (%)	52	2,28 (%)	50	2,41 (%)
M	Cell wall/membrane/envelope biogenesis	83	4,03 (%)	83	3,65 (%)	83	4,00 (%)
N	Cell motility	0	0,00 (%)	0	0,00 (%)	0	0,00 (%)
Z	Cytoskeleton	1	0,05 (%)	1	0,04 (%)	1	0,05 (%)
W	Extracellular structures	0	0,00 (%)	0	0,00 (%)	0	0,00 (%)
U	Intracellular trafficking, secretion, and vesicular transport	20	0,97 (%)	20	0,88 (%)	20	0,96 (%)
O	Posttranslational modification, protein turnover, chaperones	72	3,49 (%)	74	3,25 (%)	74	3,56 (%)
X	Mobilome: prophages, transposons	0	0,00 (%)	0	0,00 (%)	0	0,00 (%)
C	Energy production and conversion	96	4,66 (%)	101	4,44 (%)	98	4,72 (%)
G	Carbohydrate transport and metabolism	96	4,66 (%)	95	4,17 (%)	97	4,67 (%)
E	Amino acid transport and metabolism	150	7,27 (%)	148	6,50 (%)	152	7,32 (%)
F	Nucleotide transport and metabolism	66	3,20 (%)	66	2,90 (%)	66	3,18 (%)
H	Coenzyme transport and metabolism	97	4,70 (%)	105	4,61 (%)	97	4,67 (%)
I	Lipid transport and metabolism	71	3,44 (%)	71	3,12 (%)	71	3,42 (%)
P	Inorganic ion transport and metabolism	144	6,98 (%)	141	6,19 (%)	138	6,65 (%)
Q	Secondary metabolites biosynthesis, transport and catabolism	25	1,21 (%)	24	1,05 (%)	25	1,20 (%)
R	General function prediction only	0	0,00 (%)	0	0,00 (%)	0	0,00 (%)
S	Function unknown	383	18,57 (%)	390	17,13 (%)	390	18,79 (%)
-	Not assigned	272	13,19 (%)	366	16,07 (%)	315	15,17 (%)
