# Supplementary figures and images for: Genome Characterization and Probiotic Potential of Corynebacterium amycolatum Human Vaginal Isolates
Source: Microorganisms. 2022 Jan 23;10(2):249. doi: 10.3390/microorganisms10020249 (PMC8878833; doi:10.3390/microorganisms10020249)

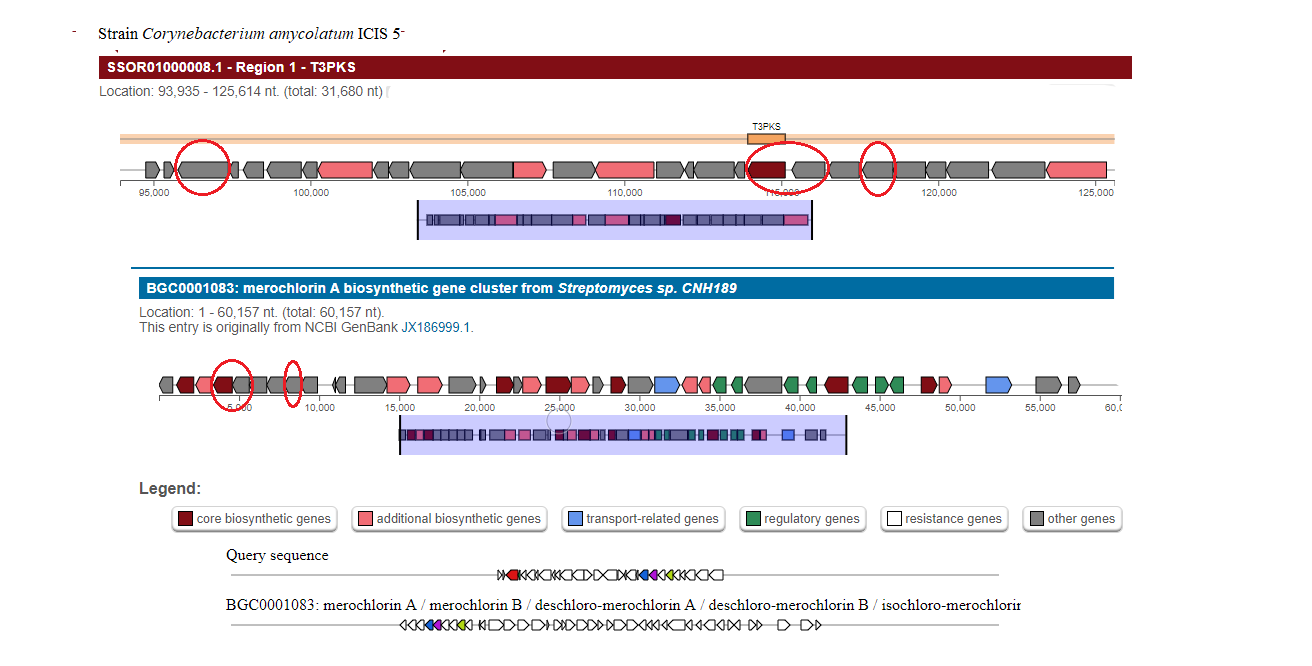

Supplement: Supplementary file 1 [file microorganisms-10-00249-s001.zip › Fig.S1.tiff]

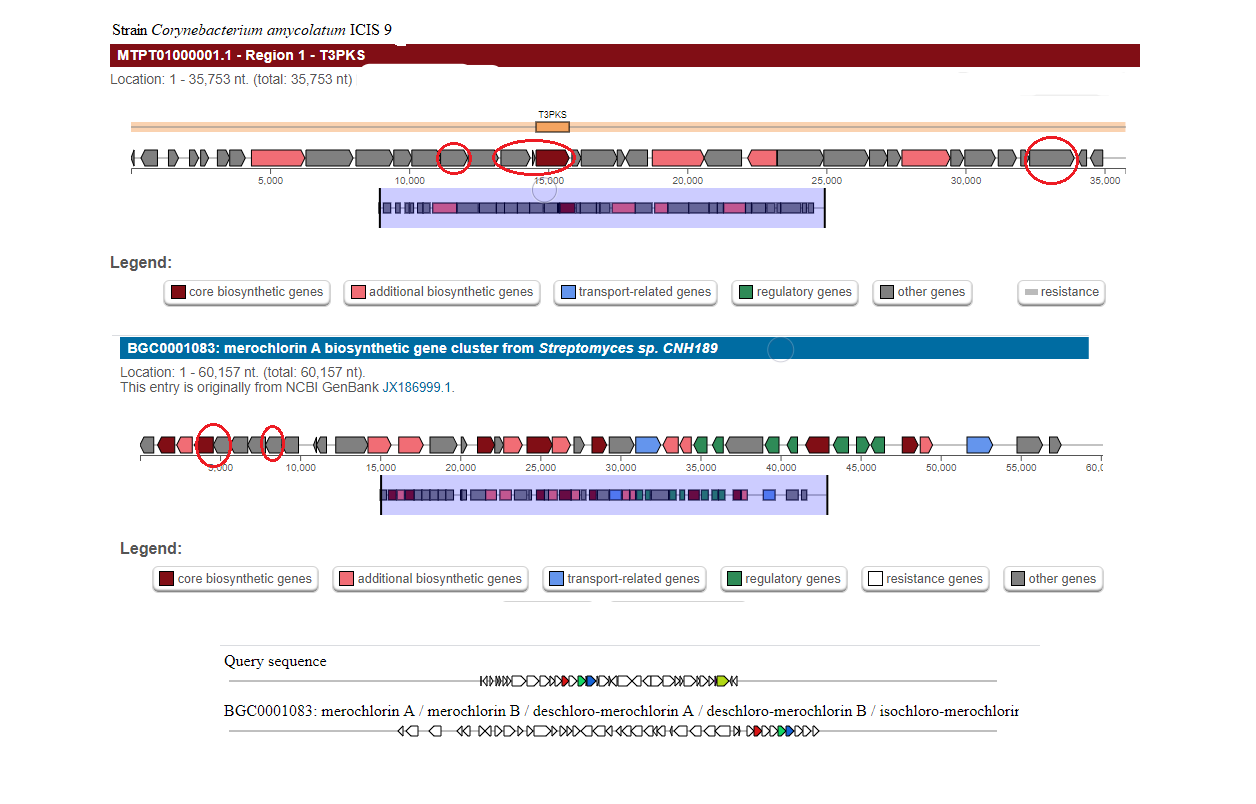

Supplement: Supplementary file 1 [file microorganisms-10-00249-s001.zip › Fig.S2.tiff]

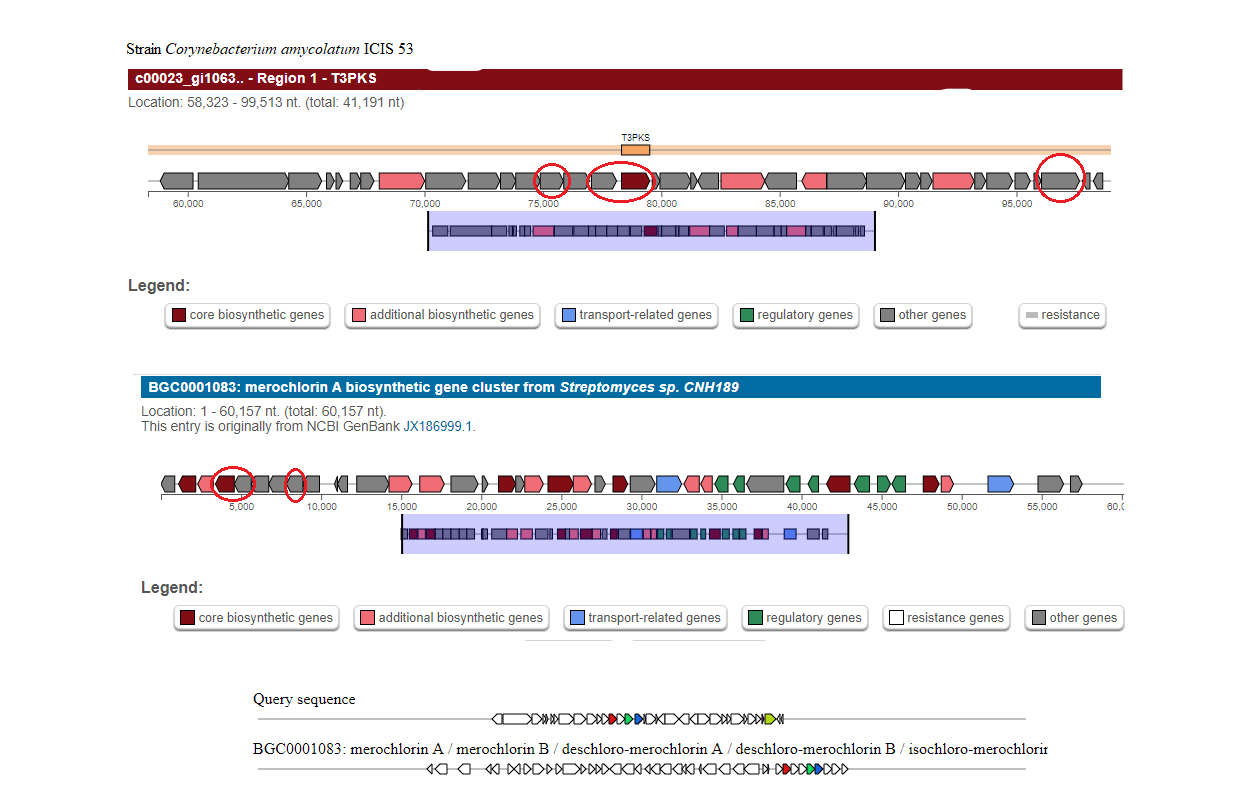

Supplement: Supplementary file 1 [file microorganisms-10-00249-s001.zip › Fig.S3.tiff]

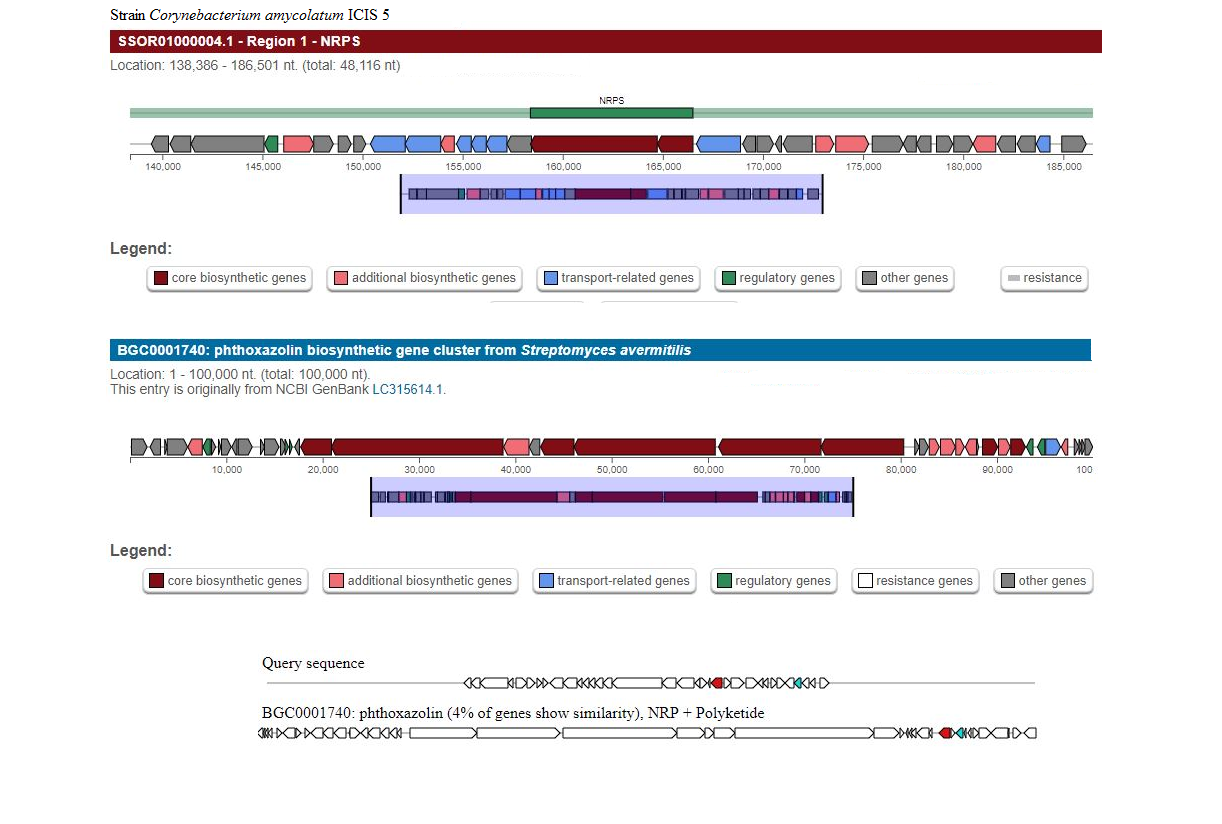

Supplement: Supplementary file 1 [file microorganisms-10-00249-s001.zip › Fig.S4.tiff]

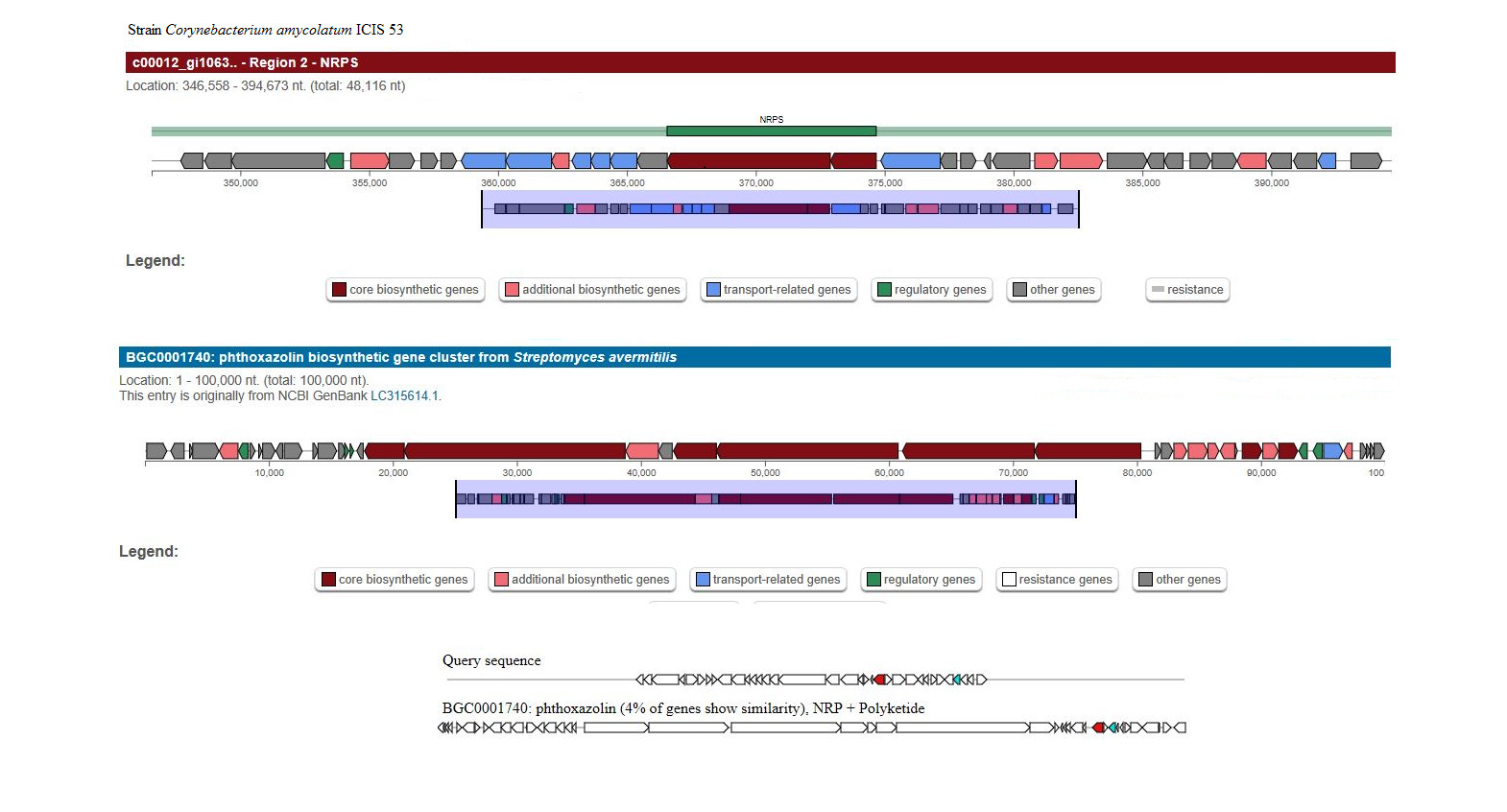

Supplement: Supplementary file 1 [file microorganisms-10-00249-s001.zip › Fig.S5.tiff]
